# Comparative evaluation of the QIAsymphony RGQ system with the easyMAG/R-gene combination for the quantitation of cytomegalovirus DNA load in whole blood

**DOI:** 10.1186/1743-422X-9-231

**Published:** 2012-10-09

**Authors:** Sylvie Pillet, Thomas Bourlet, Bruno Pozzetto

**Affiliations:** 1 Laboratory of Bacteriology-Virology-Hygiene, University Hospital of Saint-Etienne, Saint-Etienne Cedex 02, F-42055, France

**Keywords:** CMV, Viral load, Automation, Molecular biology, Real-time PCR

## Abstract

**Background:**

The detection of cytomegalovirus (CMV) DNA in blood is a key feature of the virological surveillance of immunocompromised patients.

**Methods:**

The QIAsymphony RGQ system (QIAGEN S.A.S., France) combines the extraction/distribution steps on QIAsymphony SP/AS instruments with amplification on a Rotor-Gene Q RT-PCR machine. This system was compared to a strategy combining an extraction step on the NUCLISENS easyMAG platform (bioMérieux) with the CMV R-gene kit (Argene) on 100 whole blood specimens collected from immunocompromised patients of the University Hospital of Saint-Etienne, France.

**Results:**

The overall agreement between the two strategies was 86% (kappa coefficient of 0.67); the 14 discrepant results corresponded to low DNA loads. The 62 samples found positive with both tests were correlated (Pearson r coefficient of 0.70, *P* < 0.01) despite an over quantitation of 0.25 log_10_ copies/ml with the easyMAG/Argene strategy (*P* < 0.001). Very close results were also obtained with a commercial panel of 10 samples with CMV loads ranging from 2.36 to 6.41 log_10_ copies/ml. The inter-run and intra-run variability was consistently lower with the QIAGEN platform.

**Conclusions:**

These results validate the performance of the QIAsymphony RGQ system for the routine quantitation of CMV DNA. This fully-automated platform reduces the hands-on time and improves standardization, traceability and quality control assessment.

## Background

Cytomegalovirus (CMV) can cause both early and late multi-organ disease post-transplantantation and remains one of the most important complications after allogeneic transplant. The detection of markers of CMV infection in blood is a key feature of the virological surveillance of immunocompromised patients [1]. Beside antigenemia that is labour intensive, difficult to standardize and requires an immediate analysis of the specimens, molecular methods based on quantitative real-time PCR (RT-PCR) technology are considered to be the main alternative option for diagnosis of CMV infection, allowing decisions to be made regarding both implementation of pre-emptive therapy, and monitoring response to therapy [2-5]. In nucleic acid amplification techniques, the extraction step is critical and needs to be carefully evaluated notably when different extraction methods are coupled to different PCR techniques [6].

The systematic use of molecular tools for the surveillance of immunocompromised patients needs high throughput machines able to monitor a large number of whole blood or plasma specimens. Automated systems integrating the extraction and amplification steps represent an attractive solution [6-8]. The recently commercialised QIAsymphony RGQ system (QIAGEN S.A.S., France) combines the extraction/distribution on the QIAsymphony Sample Preparation (SP) and Assay Setup (AS) modules respectively, together with the amplification step on a Rotor-Gene Q (RGQ) RT-PCR machine using the *artus* CMV QS-RGQ kit.

In this study, the fully-automated QIAsymphony RGQ system was compared to the molecular strategy currently used in our laboratory for the determination of CMV DNA load from whole blood specimens, which combines extraction with the NUCLISENS easyMAG instrument (bioMérieux, Marcy l’Etoile, France) and amplification using the CMV R-gene kit (Argene, Verniolle, France) as previously validated in our hands [9].

## Methods

### Whole blood specimens

One hundred whole blood specimens were included in the study. Samples were obtained from immunocompromised patients hospitalised at the University Hospital of Saint-Etienne, France, for the following clinical pictures scenarios: bone-marrow graft, kidney transplantation or chronic HIV infection. For all of them, the CMV DNA load was routinely evaluated as part of standard patient care. The study was conducted on the residual clinical specimens stored at −20°C. The research was approved by the Ethics Committee of the University Hospital of Saint-Etienne on the 30^th^ of May 2012. For the study, the samples were thawed, divided into 2 aliquots and tested by both systems on the same day. Specimens were then stored at 4°C until validated results of quantitation with both amplification methods (<24h).

### QCMD panel

The 2010 QCMD (Quality Control for Molecular Diagnostics) program (Glasgow, UK) included 1 negative and 9 positive (AD169 strain) specimens with viral loads ranging from 230 to 2,552,701 copies/ml, diluted either in CMV-negative human plasma or in virus transport medium. The specimens were reconstituted, separated in aliquot fractions and stored at −80°C as recommended by the QCMD instructions. After thawing, they were tested in the same way as clinical samples with both extraction and amplification systems.

### CMV DNA extraction and amplification

All the experiments were conducted with in vitro diagnosis (IVD) certified kits according to the manufacturers’ instructions.

### QIAsymphony RGQ system

Just before extraction, 300 μl volumes of the whole blood samples were manually transferred into 2-ml vials. The tubes were placed into 24-tube capacity carrier racks and loaded into the SP module. The extraction step was performed using the QIAsymphony DNA Mini Kit and the Virus Blood 200 protocol with automated eluate dispensing. The internal control of the *artus* CMV QS-RGQ kit was added into ATE buffer and automatically distributed during the lysis of samples. With this protocol, 200 μl volumes of whole blood were extracted and eluted into a total volume of 90 μl, allowing a minimum accessible volume of 60 μl. The rack containing the elution tubes was then automatically transferred to the AS module. A 30 μl volume of the PCR mix prepared by the AS module using the *artus* CMV QS-RGQ kit was automatically distributed into RG 72 strip tubes (all reagent and eluate positions are continually cooled) and after distribution of 20 μl of template by the AS module, the RG 72 strip tubes were closed and placed manually into the 72-well rotor of the Rotor-Gene Q. The amplification step was performed according to the manufacturer’s instructions. Each PCR run included a set of quantitative calibrators corresponding to 10, 100, 1000 and 10000 copies/μl of template; the CMV DNA load was calculated from the standard curve and expressed as the number of CMV DNA copies/ml of whole blood (after calculating the input and output volume). The presence of PCR inhibition was detected when the CT value for the internal control of the sample was more than 3 cycles higher than the CT value for the internal control of a negative whole blood control.

### Combination of extraction with NUCLISENS easyMAG instrument and amplification with the CMV R-gene kit

Just before extraction, 200-μl volumes of the whole blood samples were manually transferred into 4-ml cryotubes containing 2 ml of lysis buffer. The whole extraction process was performed on the NUCLISENS easyMAG instrument according to the previously validated protocol [9]. With this protocol, 200 μl of whole blood was extracted and eluted into a total volume of 50 μl. The eluates were manually transferred into 2ml-tubes. Fifteen micro liters of ready-to-use PCR mix of the CMV R-gene kit and 10 μl of template were manually distributed into a plate stored in a pre-cooled rack. The amplification step was performed using an ABI7500 instrument (Applied Biosystems) according to Argene’s instructions. Each PCR run included a set of quantitative calibrators corresponding to 5, 50, 500 and 5000 copies/μl of template; the CMV DNA load was calculated from the standard curve and expressed as the number of CMV DNA copies/ml of whole blood. The presence of PCR inhibition was detected when the CT value for the internal control of the sample was more than 3 cycles higher than the CT value for the internal control of a negative whole blood control.

### Analytic performances of the QIAGEN workflow

The analytical performances of the QIAGEN workflow were studied with frozen pooled blood samples from patients of our hospital with known CMV DNA load values. The limit of detection of the test was assessed using seven 0.5-log_10_ dilution series of CMV DNA positive whole blood in CMV DNA negative blood to yield concentrations ranging from 14 to 10000 CMV DNA copies/ml. Each measure was tested on 5 replicate aliquots.

The reproducibility of the QIAGEN system was determined in comparison with the easyMAG/Argene technique using 4 pools prepared from positive specimens and exhibiting different DNA loads: one low (< 3 log_10_ copies/ml), two intermediate (between 3 and 4.5 log_10_ copies/ml) and one high (> 4.5 log_10_ copies/ml). For each concentration, 8 frozen aliquots were tested, 4 for intra-run reproducibility assay and 4 for inter-run reproducibility assay (performed each on a different day).

### Correlation

The correlation analysis between the two systems was performed using a set of paired aliquots of whole blood specimens. The extraction and amplification steps were performed the same day for the same sample with both techniques.

### Statistical analysis

The lower limit of detection of the QIAGEN assay was determined by probit analysis [10]. The agreement between methods’ results was tested by the kappa coefficient. Bivariate correlation analysis was performed with the Pearson r coefficient and a two-tailed test of significance. Differences in mean values were analysed by the paired t test. *P* values of 0.05 were considered as the threshold of significance.

## Results

### Limit of detection and reproducibility of the QIAGEN workflow

**Figure 1:**
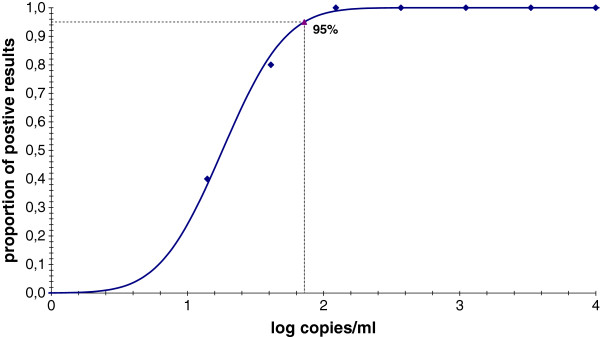
**Probit curve used to calculate the limit of detection of the QIAsymphony RGQ method.** Seven 0.5-log_10_ dilution series of CMV DNA positive whole blood in CMV DNA negative blood (lozenges) to yield concentrations ranging from 14 to 10000 CMV DNA copies/ml were tested 5 times each. The limit of detection at 95% was extrapolated from the sigmoid curve.

The limit of detection of the QIAGEN workflow, calculated by probit analysis, was 72 copies/ml, with a confidence of 95% (Figure 1). By comparison to the easyMAG/Argene system, the inter-run and intra-run variability was consistently lower with the QIAGEN platform, notably for the low and intermediate viral loads (Table 1).

**Table 1:** Intra- and inter-run reproducibility of the two strategies compared in the study on 4 replicates of each pool

	Mean (% coefficient of variation)*			
	**QIAGEN system**		**easyMAG/Argene combination**	
	**Intra-run**	**Inter-run**	**Intra-run**	**Inter-run**
Pool 1	2.30 (6.39)	2.47 (20.54)	2.82 (10.48)	2.48 (23.75)
Pool 2	3.51 (2.13)	3.53 (2.21)	4.54 (9.75)	4.00 (15.71)
Pool 3	3.70 (2.61)	3.58 (2.84)	4.34 (4.12)	4.33 (6.17)
Pool 4	4.83 (2.83)	4.72 (3.02)	5.12 (1.18)	5.12 (0.89)

* log_10_ copies/ml of whole blood.

### QCMD panel analysis

**Figure 2:**
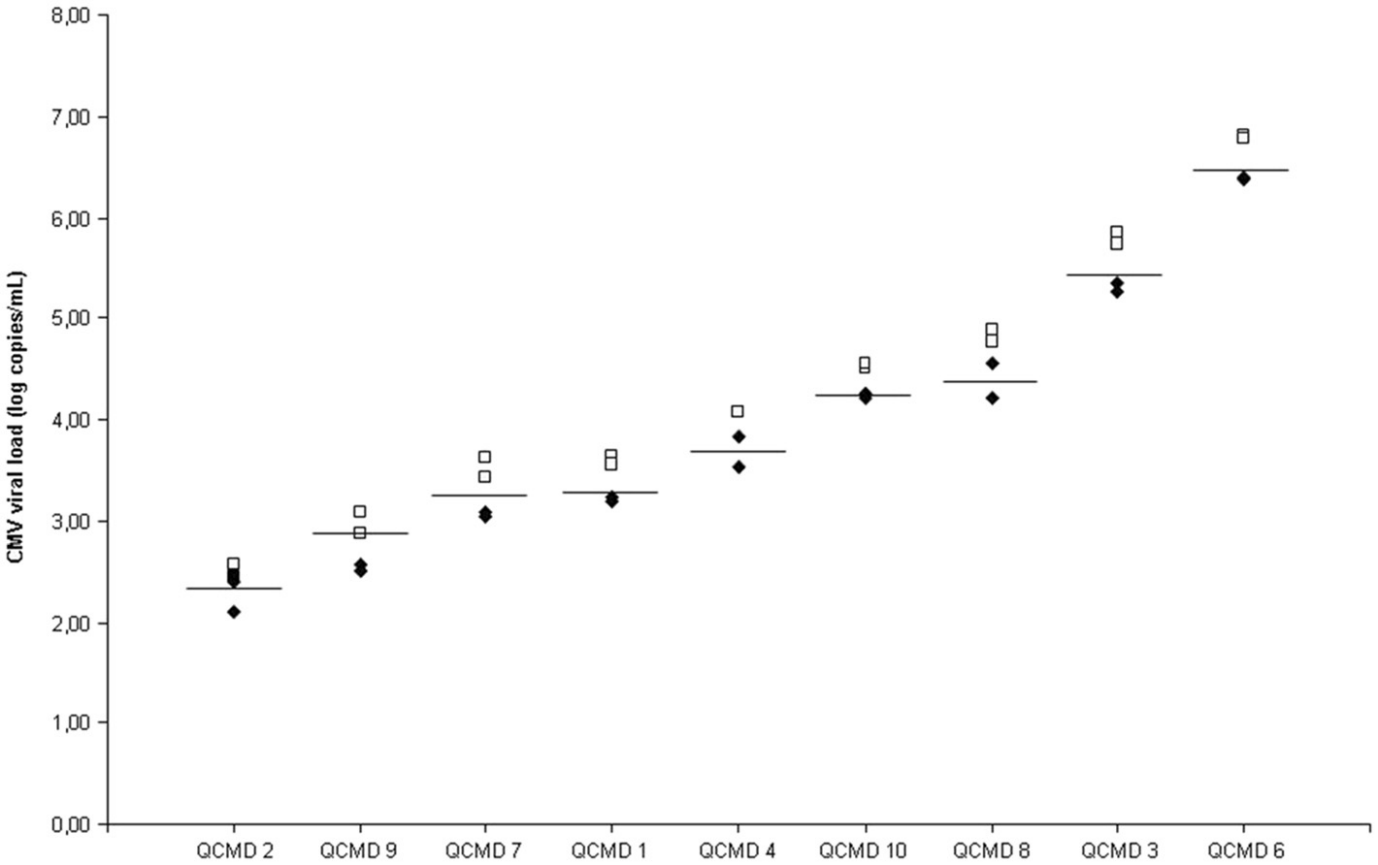
**CMV DNA load obtained with both methods for the samples of the 2010 QCMD program.** Viral loads obtained with the QIAsymphony RGQ system and the easyMAG/Argene combination are represented with diamonds and squares, respectively. The measures were performed in duplicate. The horizontal bars show the theoretical viral loads given by the manufacturer of the proficiency panel.

Ten samples belonging to the 2010 QCMD panel were tested in duplicate with the two systems. The negative sample was correctly assigned by both techniques. The results for the 9 positive samples are illustrated in Figure 2: the two systems exhibited the same range of quantitation, despite the fact that the viral load was consistently higher with the easyMAG/Argene method.

### Correlation between the test systems in clinical specimens

From the 100 clinical samples tested comparatively with both systems, 3 needed to be tested again for an invalid internal control value, 1 for easyMAG/Argene and 2 for QIAGEN and could be validated after retest.

Of the 100 pairs of clinical samples, 62 were positive with both methods and 24 were negative, which corresponds to a substantial agreement of 86% (kappa coefficient of 0.67). Fourteen samples exhibited discrepant results: 11 were tested positive with the QIAGEN platform and negative with the easyMAG/Argene method and 3 samples exhibited the opposite result; all of these discrepant results corresponded to viral loads < 2.5 log copies/ml.

**Figure 3:**
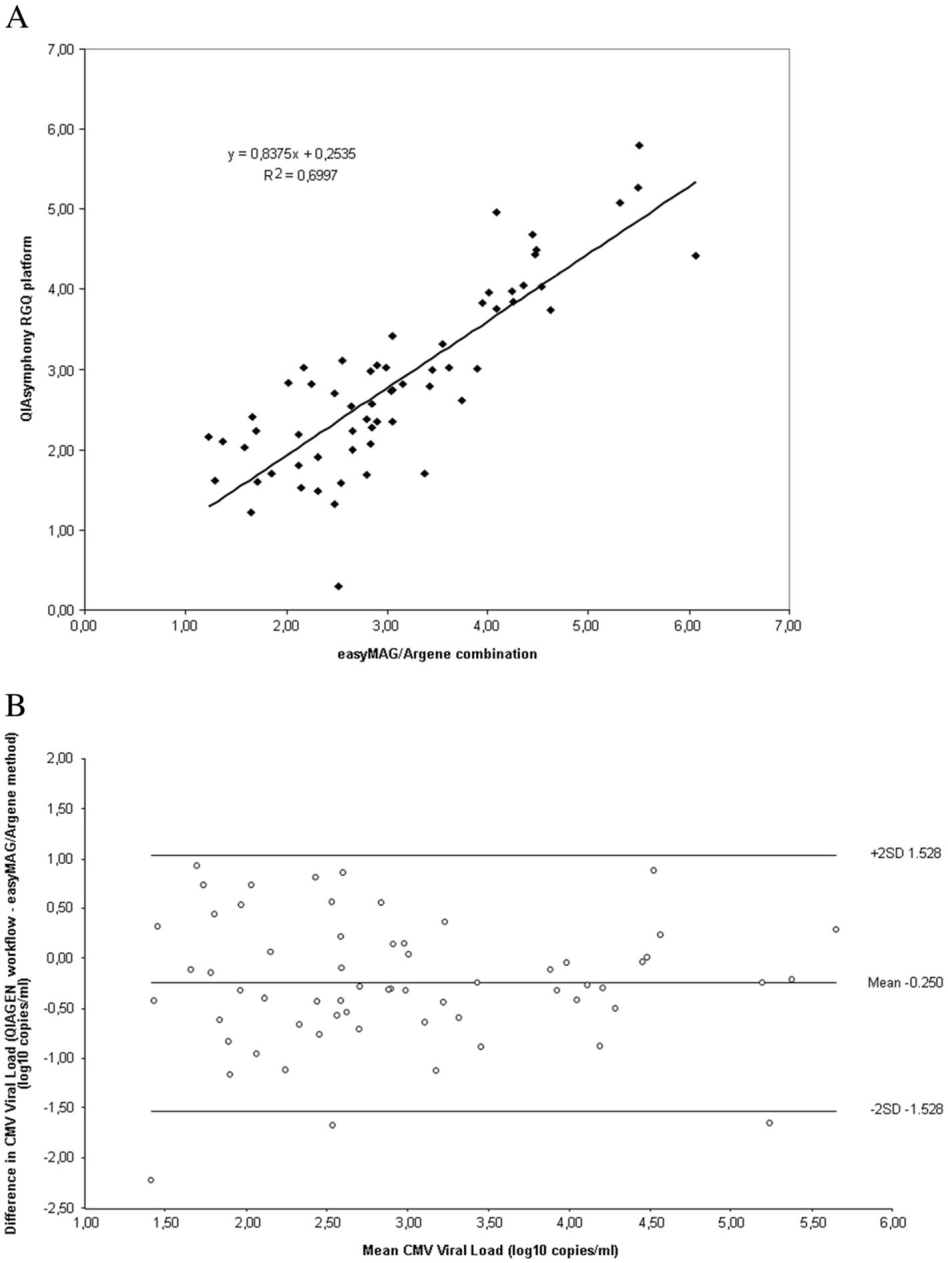
**Correlation analysis using clinical samples.** (**A**) Comparison of CMV DNA load by QIAsymphony RGQ platform and easyMAG/Argene combination using whole blood samples (N=62). The results are expressed in log_10_ copies/ml. (**B**) Difference in quantitation between QIAsymphony RGQ platform and easyMAG/Argene combination. Each point represents the difference observed between the results of the 2 methods against their mean. The standard deviation (SD) is 0.639.

The 62 samples found positive with both tests were used to draw the correlation curve (Figure 3A) and the Bland-Altman plot (Figure 3B). The Pearson r coefficient was 0.70 (*P* < 0.01) and the mean difference in viral loads was 0.25 log_10_ copies/ml to the benefit of the easyMAG/Argene strategy (*P* < 0.001). Samples corresponding to low CMV DNA loads exhibited a high variability. Among those with a CMV DNA load above 1000 copies/ml, 6 exhibited discrepancy of at least 0.5 log copies/ml: 5 with CMV load higher by the easyMAG/Argene method, one of them being below the mean of the difference – 2 SD in Figure 3B, and one with CMV load higher by the QIAGEN platform.

The time necessary to complete a run was evaluated to 3.5 and 3.7 hours for the easyMAG/Argene strategy and the QIAGEN system, respectively. However, the hands-on time was approximately of 0.3 hour for the QIAGEN system versus 1 hour for the easyMAG/Argene strategy.

## Discussion

The automation of molecular techniques performed on a large scale is an important challenge in clinical virology. The QIAGEN workstation consisting of the QIAsymphony SP and AS modules that has been recently commercialised allows the successive achievement of nucleic acid purification from various samples (SP module) and the distribution of the extract combined with master mix in PCR tubes (AS module). This automated system has been recently evaluated for the detection of enteric pathogens in faecal samples [11,12] and the quantitation of hepatitis C viral load [13]. In addition, three other studies evaluated the QIAsymphony SP extraction module alone for Epstein-Barr virus [14], HIV viral load [15] and a panel of different viruses [16].

In this study, we evaluated the QIAGEN workstation combined with the RGQ platform for the quantitation of CMV DNA with the CMV *artus* CMV QS-RGQ kit from 200 μl of whole blood. Different versions of this amplification kit have been shown to yield comparable results of CMV DNA load with other molecular techniques either in EDTA-plasma [17,18] or in whole blood [19].

Regarding the extraction step, previous studies evaluated the QIAsymphony system for the quantitation of CMV DNA in blood under different configurations: Raggam et al. [20] tested the QIAsymphony SP module in combination with the R-gene kit from 200 μl of whole blood; Miller et al. [21] evaluated the same extraction module with the Roche kit from 1 ml of blood serum; finally, Forman et al. [22] tested the same configuration as the one used in the present study but from a different matrix (1.2 ml of blood plasma). The limit of detection of these different methodologies was 148, 90, and 23 CMV DNA copies/ml, respectively; the value of 72 copies/ml obtained in the current study with the QIAsymphony RGQ system using a small volume (200 μl) of whole blood was very close to that of the three former studies and to that specified in the QIAGEN handbook of the kit (164.6 copies/ml). It was much lower than that mentioned for the R-gene technique in the manufacturer’s handbook but that had been obtained with a MagNA Pure Compact automate (555 copies/ml).

By reference to the strategy routinely used in our laboratory that we had previously evaluated favourably [9], the QIAsymphony RGQ system was well correlated, despite a slight translation of CMV DNA loads to the benefit of the easyMAG/R-gene couple. By contrast, a better sensitivity was obtained with the QIAsymphony RGQ system for low positive samples as illustrated by the distribution of positive discrepant samples (11 for the QIAGEN platform and only 3 for the easyMAG/Argene method). Furthermore, the inter-assay and intra-assay variability was shown to be lower with the QIAsymphony RGQ system because of its complete automation, whereas the easyMAG/R-gene combination includes several manual steps (sample preparation, addition of magnetic silica, transfer of eluates into microtubes, preparation and distribution of PCR mix, calibrators and samples…).

## Conclusion

In addition to fulfilling excellent technical performances (linearity comprised between 1 × 10^3^ and 5× 10^7^ copies/ml, and 95% limit of detection of 164.6 copies/ml for whole blood according to the manufacturer’s specifications), the QIAsymphony RGQ system offers a fully-automated workflow with reduction of hands-on time and improvement in standardisation, traceability and quality control assessment. It appears particularly adapted to the routine surveillance of CMV DNA load in immunocompromised patients.

## Competing interest

Except the fact that the reagents were provided free of charge by QIAGEN S.A.S., France, the authors declare no conflict of interest neither with this society nor with any other.

## Authors’ contribution

SP and BP designed the study; SP made the experiments; TB contributed to the statistical analysis and to the critical reading of the manuscript; SP and BP wrote the paper. All authors read and approved the final manuscript.
